# The Wnt/β-Catenin Pathway Interacts Differentially with PTHrP Signaling to Control Chondrocyte Hypertrophy and Final Maturation

**DOI:** 10.1371/journal.pone.0006067

**Published:** 2009-06-26

**Authors:** Xizhi Guo, Kinglun Kingston Mak, Makoto M. Taketo, Yingzi Yang

**Affiliations:** 1 Developmental Genetics Section, National Human Genome Research Institute, Bethesda, Maryland, United States of America; 2 Bio-X Center, Shanghai Jiao Tong University, Haoran Building, Shanghai, People's Republic of China; 3 Department of Pharmacology, Kyoto University Graduate School of Medicine, Yoshida-Konoé-cho, Sakyo-ku, Kyoto, Japan; Hopital Cochin, France

## Abstract

Sequential proliferation, hypertrophy and maturation of chondrocytes are required for proper endochondral bone development and tightly regulated by cell signaling. The canonical Wnt signaling pathway acts through β-catenin to promote chondrocyte hypertrophy whereas PTHrP signaling inhibits it by holding chondrocytes in proliferating states. Here we show by genetic approaches that chondrocyte hypertrophy and final maturation are two distinct developmental processes that are differentially regulated by Wnt/β-catenin and PTHrP signaling. Wnt/β-catenin signaling regulates initiation of chondrocyte hypertrophy by inhibiting PTHrP signaling activity, but it does not regulate *PTHrP* expression. In addition, Wnt/β-catenin signaling regulates chondrocyte hypertrophy in a non-cell autonomous manner and Gdf5/Bmp signaling may be one of the downstream pathways. Furthermore, Wnt/β-catenin signaling also controls final maturation of hypertrophic chondrocytes, but such regulation is PTHrP signaling-independent.

## Introduction

Mammalian skeletal development is controlled by two mechanisms, endochondral and intramembranous bone formation. Endochondral ossification occurs in most parts of the body and requires a cartilage model prior to bone formation. Cartilage is composed of chondrocytes and in the developing long bones, these chondrocytes are organized into zones with distinct cellular morphologies and proliferation properties. Such organization is essential for the proper directional growth and elongation and is subject to tight regulation by secreted signaling molecules and transcription factors [1]–[3]. Proliferating chondrocytes are located at both ends of the cartilage and express *Sox9* and *Col2a1*. Closer to the middle of the cartilage, proliferating chondrocytes become elongated and line up in columns along the longitudinal axis. These columnar chondrocytes divide quickly and begin to express Indian hedgehog (*Ihh*) and *Runx2* as they exit the cell cycle to become prehypertrophic chondrocytes. Prehypertrophic chondrocytes then differentiate into hypertrophic chondrocytes that no longer express *Sox9* and *Col2a1*, but express *Col10a1* and angiogenic factors such as vascular endothelial growth factor (*Vegf*). Hypertrophic chondrocyes can be further divided into two populations according to their differences in gene expression and mineralization levels. *Ihh* and parathyroid hormone receptor 1 (*Pthr1*) are expressed only in prehypertrophic and early hypertrophic chondrocytes. The mature population of hypertrophic chondrocytes express Osteopontin (*Opn*) [4] and matrix metalloproteinase 13 (*Mmp13*) that is required for invasion of blood vessels and osteoblasts to form the trabecular bone [5]. These most mature hypertrophic chondrocytes undergo apoptosis and are removed eventually. In addition, calcium deposition in the cartilage, which can be visualized by Von Kossa and Alizarin red staining, only occurs in the extracellular matrix of the final mature hypertrophic chondrocytes. Thus, both chondrocyte hypertrophy and final maturation are required for endochondral bone formation. However, it is not clear whether chondrocyte hypertrophy and final maturation are two consecutive developmental processes regulated by the same molecular pathways or they are actually developmentally separated and differentially regulated.

Ihh, parathyroid hormone related peptide (PTHrP), Wnts, Bone morphogenetic proteins (Bmps) and fibroblast growth factor (Fgfs) etc., regulate progressive chondrocyte proliferation and hypertrophy. PTHrP and Ihh are major regulators of chondrocyte hypertrophy by forming a negative feedback loop [6]. *Ihh* activates *PTHrP* expression and PTHrP then signals to proliferating chondrocytes to inhibit *Ihh* expression and chondrocyte hypertrophy by holding chondrocytes in a proliferating state. Wnt signaling pathways also regulate chondrocyte hypertrophy [7]–[10]. Among known Wnt signaling pathways, the canonical Wnt signaling pathway mediated by β-catenin [11] is the best understood and has been found to promote both chondrocyte hypertrophy and final maturation [9], [10]. Wnt/β-catenin signaling acts independently of Ihh signaling to promote chondrocyte hypertrophy [12], but it is unknown whether Wnt/β-catenin and PTHrP signaling pathways regulate each other to control chondrocyte hypertrophy and maturation.

Here we have investigated the regulation of chondrocyte hypertrophy and final maturation and the genetic relationship between the canonical Wnt and PTHrP signaling pathways in sequential chondrocyte differentiation. We uncovered that chondrocyte hypertrophy and final maturation are two distinct processes that are differentially regulated by Wnt/β-catenin and PTHrP signaling. Canonical Wnt signaling promotes chondrocyte hypertrophy by antagonizing PTHrP signaling activity. However, the final maturation of hypertrophy chondrocytes is controlled by Wnt/β-catenin signaling independently of PTHrP signaling.

## Results

### Wnt signaling controls chondrocyte hypertrophy by antagonizing PTHrP signaling

**Figure 1:**
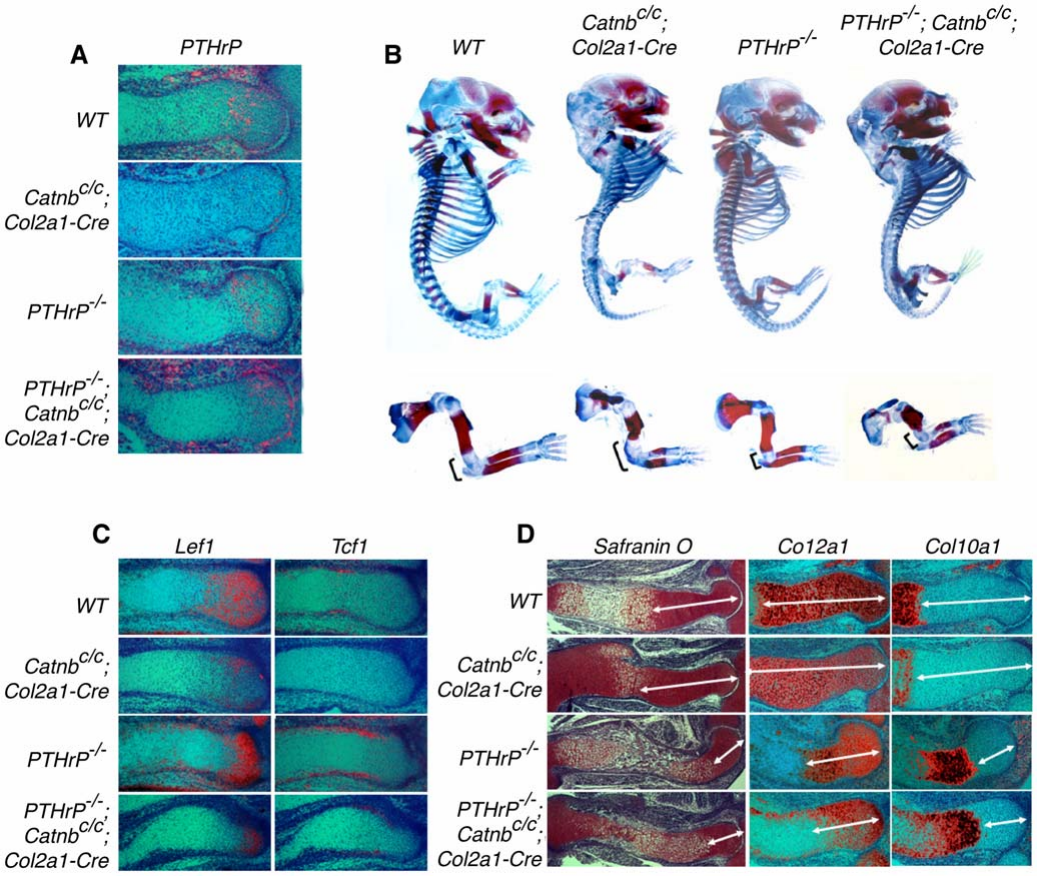
Analysis of *PTHrP* and *β-catenin* mutant skeletons. (A) Sections of developing humerus at 14.5 dpc were examined by in situ hybridization with *PTHrP* probes. Expression of *PTHrP* was reduced in periarticular chondrocytes in *Catnb^c/c^; Col2a1-Cre* single mutant embryos compared to that in wild type embryos. *PTHrP* expression was increased in the double mutant embryos compared to that in the *Catnb^c/c^; Col2a1-Cre* single mutant embryos. (B) Skeletal preparation of 16.5 dpc embryos. A forelimb of each embryo is shown in lower panel. Alizarin red stains the mineralized hypertrophic chondrocytes and bone. Alcian blue stains the unmineralized cartilage. Mineralization was accelerated in the *PTHrP^−/−^; Catnb^c/c^; Col2a1-Cre* double mutant and the *PTHrP^−/−^* single mutant. The bracket indicates the non-mineralized cartilage at the joint region. (C) Consecutive sections of the radius at 14.5 dpc were examined by in situ hybridization with probes of *Lef1* and *Tcf1*. *Lef1* and *Tcf1* expression were downregulated in both *Catnb^c/c^; Col2a1-Cre* single mutant and *PTHrP^−/−^; Catnb^c/c^; Col2a1-Cre* double mutant embryos. (D) Consecutive sections of the developing humerus at 14.5 dpc were examined by Safranin O staining and in situ hybridization with the indicated riboprobes. Safranin O staining and expression of *Col2a1* marked nonhypertrophic chondrocytes. The nonhypertrophic domain (double headed arrow) was expanded in *Catnb^c/c^; Col2a1-Cre* mutant embryos whereas it was shortened to the same degree in *PTHrP^−/−^* single mutant and *PTHrP^−/−^; Catnb^c/c^; Col2a1-Cre* double mutant embryos. *Col10a1* expression marked hypertrophic region, which was accelerated to the same level in *PTHrP^−/−^* single mutant and *PTHrP^−/−^; Catnb^c/c^; Col2a1-Cre* double mutant embryos.

To test whether Wnt/β-catenin signaling acts through the PTHrP pathway in regulating chondrocyte hypertrophy, we first examined whether *PTHrP* expression was regulated by Wnt/β-catenin signaling in the developing long bone cartilage. *β-catenin* was removed in chondrocytes using the *Col2a1-Cre* mouse line [13]. The onset of chondrocyte hypertrophy at 12.5 days post coitum (dpc) were not altered [9]. However, continued initiation of chondrocyte hypertrophy by the remaining proliferating chondrocytes was slower in the *Catnb^c/c^;Col2a1-Cre* mouse embryos [9]. *PTHrP* expression in the *β-catenin* deficient cartilage was similar to that in the control, but weaker at 14.5dpc (Fig. 1A). This is likely secondary to weakened *Ihh* expression in the cartilage of the *Catnb^c/c^; Col2a1-Cre* mouse embryo [9], [14], since ligand-independent activation of Hedgehog (Hh) signaling in the *Catnb^c/c^; Col2a1-Cre* mice rescues *PTHrP* expression [12]. Furthermore, if Wnt/β-catenin signaling acts by regulating *PTHrP* expression to control chondrocyte hypertrophy, reduced *PTHrP* expression should lead to accelerated chondrocyte hypertrophy, the opposite of the observed phenotype for the *Catnb^c/c^*; *Col2a1-Cre* mouse embryo [9], [10]. These data suggest that Wnt/β-catenin signaling regulates chondrocyte hypertrophy independently of *PTHrP* expression.

The possibility remains that β-catenin interacts with PTHrP signaling in PTHrP-responding chondrocytes. To test this, we generated the *PTHrP^−/−^; Catnb^c/c^; Col2a1-Cre* double mutant mouse embryos. If Wnt/β-catenin signaling acts downstream of PTHrP signaling, initiation of chondrocyte hypertrophy should be similarly delayed in both the *Catnb^c/c^; Col2a1-Cre* and *PTHrP^−/−^; Catnb^c/c^; Col2a1-Cre* embryos compared to that in the wild type embryo. Conversely, if Wnt/β-catenin signaling acts upstream of PTHrP signaling, initiation of chondrocyte hypertrophy should be similarly accelerated in the *PTHrP^−/−^* and *PTHrP^−/−^; Catnb^c/c^; Col2a1-Cre* embryos. Single and double mutant embryos were harvested together with their wild type control littermates and analyzed by skeletal preparations. The relative length of Alizarin red stained segment (shows mineralized cells) versus Alcian blue stained segment (shows unmineralized chondrocytes) was compared in the same cartilage elements (Fig. 1B). Long bone mineralization was increased in the *PTHrP^−/−^* single mutant, but reduced in the *Catnb^c/c^; Col2a1-Cre* and *PTHrP^−/−^; Catnb^c/c^; Col2a1-Cre* embryos (Fig. 1B). These results suggest that final maturation of hypertrophic chondrocytes was similarly delayed in the *Catnb^c/c^; Col2a1-Cre* and *PTHrP^−/−^; Catnb^c/c^; Col2a1-Cre* double mutant embryos.

Initiation and final maturation of chondrocyte hypertrophy were then examined more closely on histological sections of the limb at 14.5 dpc and 16.5 dpc. In both *Catnb^c/c^; Col2a1-Cre* and *PTHrP^−/−^; Catnb^c/c^; Col2a1-Cre* double mutant embryos, Wnt signaling activity was similarly reduced as shown by the reduction in the expression of two transcriptional targets of canonical Wnt signaling, *Lef1* and *Tcf1* (Fig. 1C) [15], [16]. Chondrocyte hypertrophy was then analyzed by Safranin O staining. Hypertrophic chondrocytes were those enlarged and lightly stained chondrocytes whereas proliferating chondrocytes were smaller and stained bright red. Surprisingly, although chondrocyte hypertrophy was delayed in the *Catnb^c/c^; Col2a1-Cre* embryo, loss of PTHrP signaling leads to an acceleration of chondrocyte hypertrophy in the *PTHrP^−/−^; Catnb^c/c^; Col2a1-Cre* embryo similar to that in the *PTHrP^−/−^* embryo (Fig. 1D). These morphological observations were further confirmed by molecular markers analysis. *Col10a1* is expressed in hypertrophic chondrocytes whereas *Col2a1* is expressed in proliferating chondrocytes. *Col2a1* expression domain was reduced whereas *Col10a1* expression domain was expanded to the same degree in the *PTHrP^−/−^* and *PTHrP^−/−^; Catnb^c/c^; Col2a1-Cre* embryos at 14.5 dpc (Fig. 1D). Similar acceleration of chondrocyte hypertrophy in *PTHrP^−/−^* and *PTHrP^−/−^; Catnb^c/c^; Col2a1-Cre* embryos was also observed in other skeletal elements such as the vertebral bodies (Fig. S1). These results indicate that Wnt/β-catenin signaling is dependent on PTHrP signaling in regulation of chondrocyte hypertrophy.

**Figure 2:**
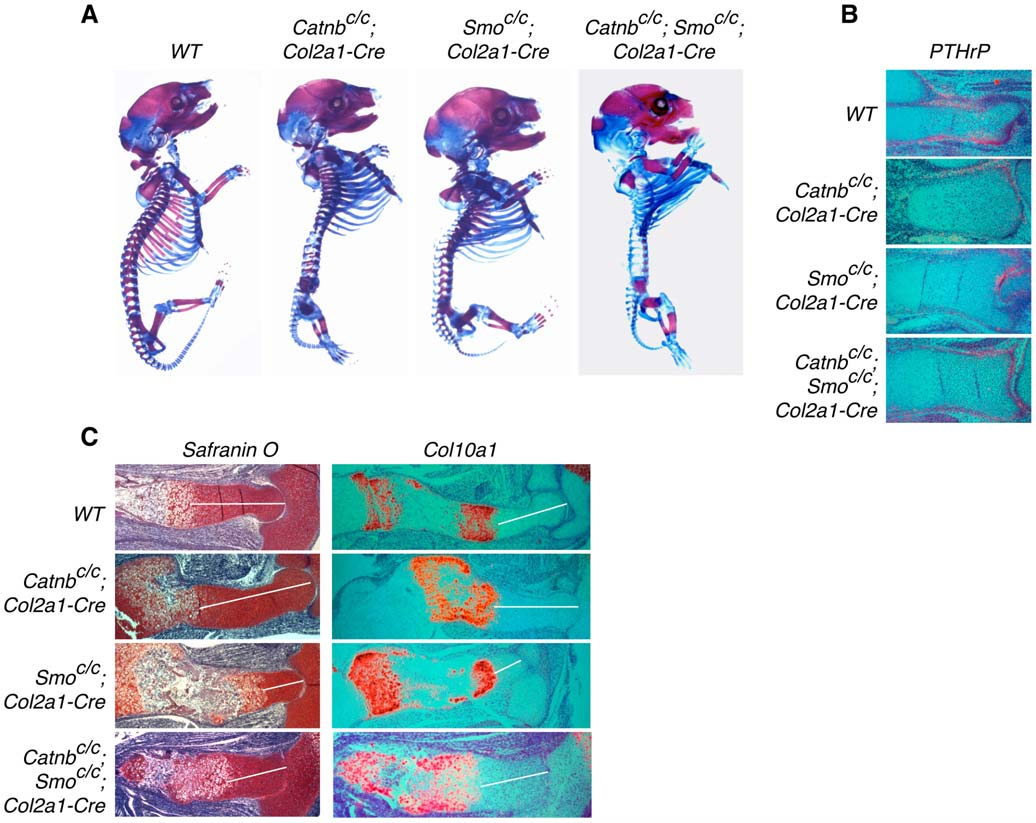
Analysis of chondrocyte hypertrophy in *β-catenin* and *Smo* mutant embryos. (A) Skeletal preparation of 18.5 dpc embryos. Mineralization (stained by Alizarin red) in the *Catnb^c/c^; Smo^c/c^; Col2a1-Cre* double mutant embryo was more advanced than that in the *Catnb^c/c^; Col2a1-Cre* mutant embryo, but less advanced than that in the *Smo^c/c^; Col2a1-Cre* mutant embryo. (B) Sections of the developing distal tibia at 15.5 dpc were examined by in situ hybridization with *PTHrP* probes. *PTHrP* expression was slightly reduced only in the periarticular chondrocytes in *Catnb^c/c^; Col2a1-Cre* mutant embryos compared to that in wild type embryos. *Catnb^c/c^; Smo^c/c^; Col2a1-Cre* and *Smo^c/c^; Col2a1-Cre* embryos have similar expression of *PTHrP*. (C) Safranin O staining and *Col10a1* expression in the developing humerus at 15.5 dpc. Non-hypertrophic chondrocyte region (white line) was expanded in the *Catnb^c/c^; Col2a1-Cre* mutant, but reduced in the *Smo^c/c^; Col2a1-Cre* mutant embryo, compared that in the wild type control. Chondrocyte hypertrophy in the *Catnb^c/c^; Smo^c/c^; Col2a1-Cre* double mutant embryo was delayed than that in the *Smo^c/c^; Col2a1-Cre* mutant embryo, but accelerated than that in the *Catnb^c/c^; Col2a1-Cre* mutant embryo.

The above data suggest that active PTHrP signaling is required for Wnt/β-catenin signaling to regulate chondrocyte hypertrophy, but PTHrP signaling may not require β-catenin to regulate chondrocyte hypertrophy. This predicts that in the absence of *β-catenin*, PTHrP signaling levels can still determine the rate of chondrocyte hypertrophy. To test this, we need to partially reduce PTHrP signaling by reducing *PTHrP* expression. In this case, *β-catenin* removal should still enhance the residual PTHrP signaling, which will lead to a delay in chondrocyte hypertrophy. To achieve these genetic manipulations, we partially reduced *PTHrP* expression by partially reducing Ihh signaling as Ihh controls *PTHrP* expression. Ihh signaling was reduced by removing *Smoothened* (*Smo*), which encodes the signaling receptor for Ihh, in the cartilage in the *Smo^c/c^; Col2a1-Cre* embryo [17] and Fig. 2A). *PTHrP* expression was diminished in the cartilage, but still detectable in the joint region in the *Smo^c/c^; Col2a1-Cre* embryo (Fig. 2B). Chondrocyte hypertrophy was accelerated in the *Smo^c/c^; Col2a1-Cre* embryo, as indicated by both Safranin O staining and analysis of *Col2a1* and *Col10a1* expression (Fig. 2C). When *β-catenin* was also removed in the *Smo^c/c^; Catnb^c/c^; Col2a1-Cre* double mutant embryo, chondrocyte hypertrophy was delayed compared to that in the *Smo^c/c^; Col2a1-Cre* embryos (Fig. 2C), despite *PTHrP* expression levels being similarly reduced in both (Fig. 2B). In addition, we observed previously that when *PTHrP* expression was similarly upregulated in *Ptch1^c/−^; Col2a1-Cre* and *Ptch1^c/−^; Catnb^c/−^; Col2a1-Cre* embryos, there was a further delay of chondrocyte hypertrophy when *β-catenin* is removed [12]. Thus, in the absence of *β-catenin*, PTHrP signaling still inhibits chondrocyte hypertrophy in a dose-dependent manner, whereas in the absence of PTHrP signaling, β-catenin has no effect on chondrocyte hypertrophy.

Delayed chondrocyte hypertrophy can be secondary to increased proliferation [18], [19]. Interestingly, chondrocyte proliferation was slightly increased in the *PTHrP^−/−^; Catnb^c/c^; Col2a1-Cre* embryos compared to that in the *PTHrP^−/−^* single mutants at 16.5 dpc (Fig. S2), although chondrocyte hypertrophy was similar in both mutants. These results indicate that Wnt/β-catenin signaling can affect the rate at which cells divide in the proliferating chondrocyte pool, but only PTHrP signaling controls the brake that determines when chondrocytes exit cell cycle to undergo hypertrophy.

### Wnt/β-catenin signaling is required for maturation of hypertrophic chondrocytes independently of PTHrP signaling

**Figure 3:**
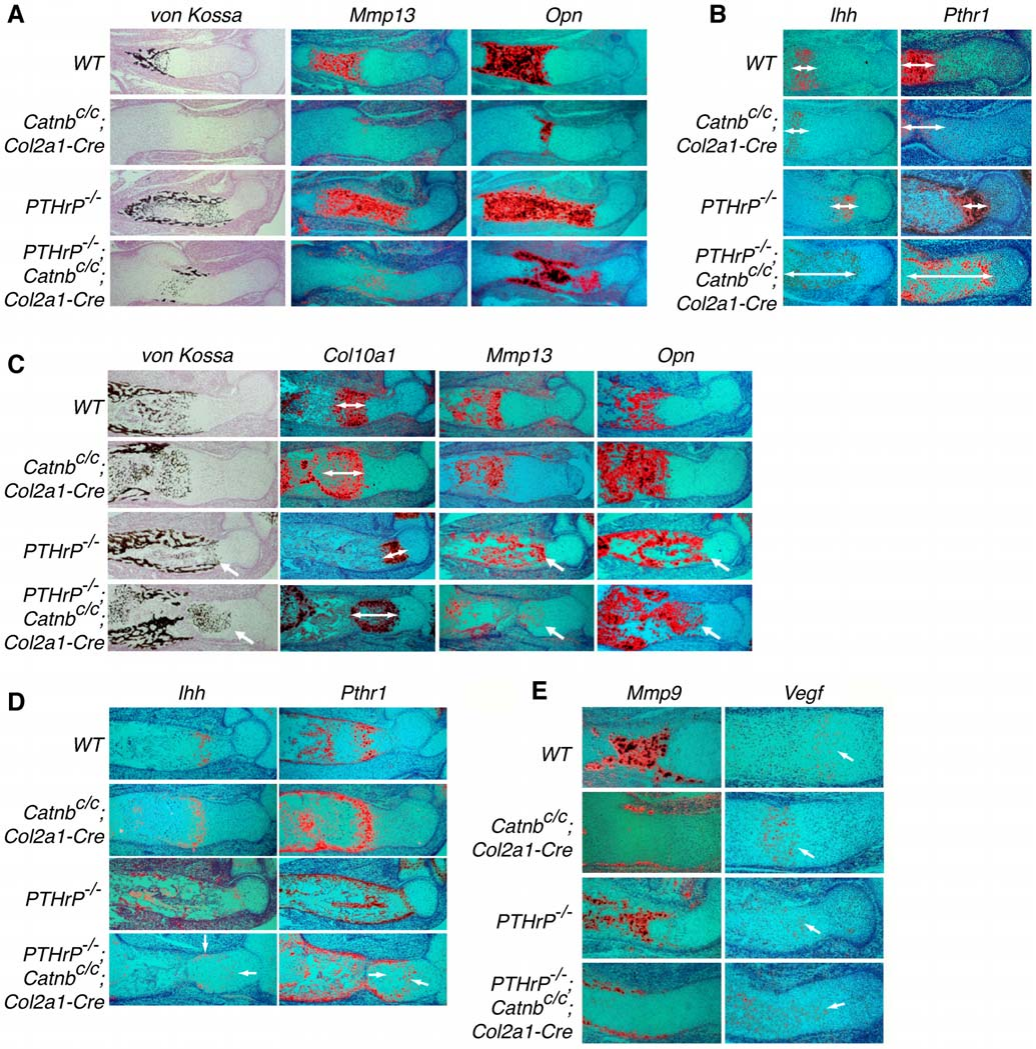
Hypertrophic chondrocyte maturation was regulated by β-catenin independently of *PTHrP*. Consecutive sections of the developing humerus at 14.5 dpc and 16.5 dpc were examined by Von Kossa staining and in situ hybridization with the indicated riboprobes. Mature hypertrophic chondrocytes were stained black by the Von Kossa method and expressed *Mmp13* and *Opn*. (A) Von Kossa staining and *Mmp13* and *Opn* expression were much reduced in both *Catnb^c/c^; Col2a1-Cre* and *PTHrP^−/−^; Catnb^c/c^; Col2a1-Cre* mutant embryos at 14.5 dpc. (B) *Ihh* and *Pthr1* were expressed in the less mature hypertrophic chondrocytes (double-headed arrow). This domain was expanded significantly only in the *PTHrP^−/−^; Catnb^c/c^; Col2a1-Cre* mutant embryos at 14.5 dpc. (C) At 16.5 dpc, chondrocyte final maturation in the *Catnb^c/c^; Col2a1-Cre* and *PTHrP^−/−^; Catnb^c/c^; Col2a1-Cre* mutant was similar to that of the wild type control and *PTHrP^−/−^* mutant, respectively. (D) Expansion of *Ihh* and *Pthr1* expression domains (white line) were still observed in the *PTHrP^−/−^; Catnb^c/c^; Col2a1-Cre* mutant at 16.5 dpc. (E) Expression of *Mmp9* and *Vegf* in the developing humerus of the indicated genotypes at 14.5 dpc.

In the normal process of long bone development, chondrocyte hypertrophy is followed by hypertrophic chondrocyte maturation to allow the invasion of osteoblasts and blood vessels. Our observation that mineralization, but not hypertrophy, was similarly delayed in the *PTHrP^−/−^; Catnb^c/c^; Col2a1-Cre* and *Catnb^c/c^; Col2a1-Cre* embryos led us to think that formation of the early and late populations of hypertrophic chondrocytes are differentially regulated. To test this, we further examined chondrocyte maturation on histological sections. At 14.5 dpc, although *Col10a1* expression domains were comparable between *PTHrP^−/−^* and *PTHrP^−/−^; Catnb^c/c^; Col2a1-Cre* embryos (Fig. 1D), in stark contrast to *PTHrP^−/−^* embryo, where maturation of hypertrophic chondrocytes was also accelerated, hypertrophic chondrocyte maturation detected by von Kossa staining was significantly delayed in the *PTHrP^−/−^; Catnb^c/c^; Col2a1-Cre* embryos (Fig. 3A). We then performed in situ hybridizations with probes specific to distinct stages of chondrocyte hypertrophy and maturation. At 14.5 dpc, expression domains of *Mmp13* and *Opn* were much smaller in the *PTHrP^−/−^; Catnb^c/c^; Col2a1-Cre* embryos compared to those in the *PTHrP^−/−^* embryos (Fig. 3A). By contrast, expression domains of *Ihh* and *Pthr1*, which mark prehypertrophic chondrocytes and early hypertrophic chondocytes, were expanded in the *PTHrP^−/−^; Catnb^c/c^; Col2a1-Cre* embryos compared to that in the *PTHrP^−/−^* embryos (Fig. 3B). Thus, in the *PTHrP^−/−^; Catnb^c/c^; Col2a1-Cre* embryo, loss of PTHrP signaling accelerated chondrocyte hypertrophy, but these hypertrophic chondrocytes were not able to undergo further maturation due to the blockage of Wnt/β-catenin signaling. As a result, the *Ihh* and *Pthr1* expressing early hypertrophic chondrocyte population was significantly expanded. This increased *Ihh* expression domain in the *PTHrP^−/−^; Catnb^c/c^; Col2a1-Cre* embryo likely caused increased osteoblast differentiation compared to that in the *Catnb^c/c^; Col2a1-Cre* embryo (Fig. 3A and S3).

By 16.5 dpc, hypertrophic chondrocyte maturation, as indicated by von Kossa staining and expression of *Mmp13* and *Opn*, caught up in the *PTHrP^−/−^; Catnb^c/c^; Col2a1-Cre* embryos with those in the *PTHrP^−/−^* embryos (Fig. 3C). However, at this stage more *Col10a1*-expressing hypertrophic chondrocytes were observed in the *Catnb^c/c^; Col2a1-Cre* and *PTHrP^−/−^; Catnb^c/c^; Col2a1-Cre* embryos compare to the wild type control and the *PTHrP^−/−^* embryos, respectively (Fig. 3C). This is likely due to reduced elimination of mature hypertrophic chondrocytes caused by delayed blood vessel invasion and bone formation when *β-catenin* was removed [9]. Consistent with this, mineralized hypertrophic chondrocytes domains were increased in both *Catnb^c/c^; Col2a1-Cre* and *PTHrP^−/−^; Catnb^c/c^; Col2a1-Cre* embryos compared to the wild type control and *PTHrP^−/−^* embryos, respectively (Fig. 3C). In addition, expression of *Mmp9*, which is a marker for osteoclasts and chondroclasts, was delayed in both *Catnb^c/c^; Col2a1-Cre* and *PTHrP^−/−^; Catnb^c/c^; Col2a1-Cre* embryos compared to the wild type control and *PTHrP^−/−^* embryos, respectively (Fig. 3E). However, *Vegf* expression, which was detected in hypertrophic chondrocytes was not decreased in *Catnb^c/c^; Col2a1-Cre* and *PTHrP^−/−^; Catnb^c/c^; Col2a1-Cre* embryos (Fig. 3E). There was also no significant change in RANKL expression (Fig. S4). These results suggest that delayed blood vessel invasion and trabecular bone formation in the *PTHrP^−/−^; Catnb^c/c^; Col2a1-Cre* embryo are at least in part a result of reduced expression of *Mmps*, not *Vegf* or RANKL. Interestingly, domains of *Ihh/Pthr1* expressing- early hypertrophic chondrocytes were still expanded in the *PTHrP^−/−^; Catnb^c/c^; Col2a1-Cre* embryos at 16.5 dpc, particularly in the periphery of the cartilage compared to that in the *PTHrP^−/−^* embryos (Fig. 3D), which created a zone with a mixture of early and late mature hypertrophic chondrocytes. Taken together, these results indicate that the Wnt/β-catenin pathway regulates hypertrophic chondrocyte final maturation, at least partly and transiently, in a manner independent of PTHrP signaling.

### Wnt signaling may regulate chondrocyte hypertrophy and maturation non-cell autonomously

**Figure 4:**
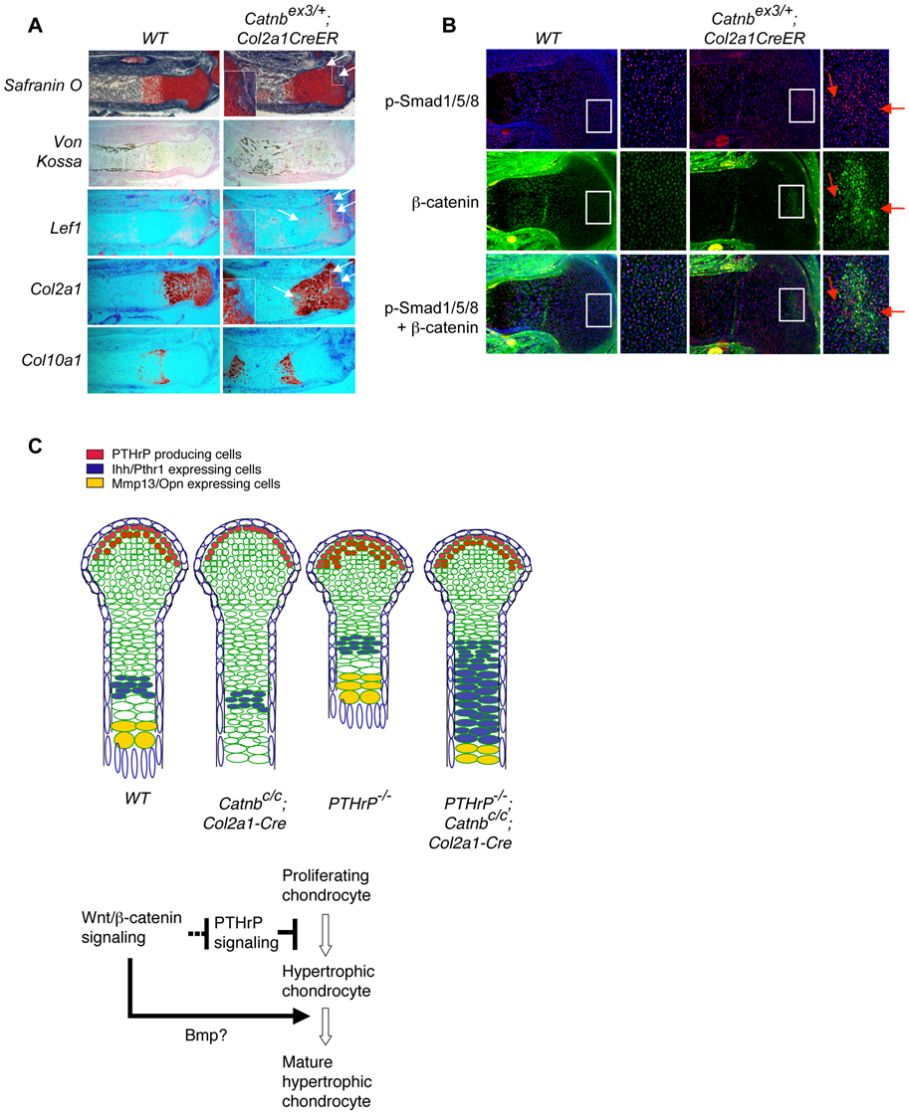
β-catenin may control chondrocyte hypertrophy non-cell autonomously. Tamoxifen was injected into pregnant females at 13.5 dpc. Embryos were harvested at 17.5 dpc and sections of the proximal tibia were analyzed by Safranin O staining, in situ hybridization with the indicated probes and immnohistochemistry. (A) Upregulated Wnt/β-catenin signaling was indicated by ectopic *Lef1* expression and loss of Safranin O staining and *Col2a1* expression (arrow). Pictures of higher magnification of the boxed area are shown as insets. *Col10a1* expression was not ectopically detected in chondrocytes that had lost *Col2a1* expression. (B) Increased p-Smad1/5/8 staining (red) was observed in chondrocyte patches of *Catnb^ex3/+^; Col2a1-CreER* mouse embryos. Such increase in p-Smad1/5/8 staining was in the area with increased β-catenin staining (green). But p-Smad1/5/8 and β-catenin staining did not colocalize in many chondrocytes. Some cells with increased p-Smad1/5/8 staining (arrows) did not show increased β-catenin staining. (C) Distinct genetic interactions between Wnt/β-catenin and PTHrP signaling in regulating chondrocyte hypertrophy and maturation. The sequential process of chondrocyte hypertrophy and maturation in developing wild type and indicated mutant long bone cartilage were shown in the diagram in the upper panel. Hypertrophic chondrocytes are enlarged and undergo final maturation before they are replaced by the trabecular bone. Initiation of chondrocyte hypertrophy and final maturation are two separate processes that are differentially regulated by Wnt/β-catenin and PTHrP signaling. Wnt/β-catenin signaling controls chondrocyte hypertrophy by inhibiting PTHrP signaling activity. However, Wnt/β-catenin signaling promoted final maturation of hypertrophic chondrocyte independently of PTHrP signaling.

As PTHrP signaling controls chondrocyte hypertrophy cell autonomously [23], we tested whether the Wnt/β-catenin signaling pathway also acts cell autonomously. We expressed an activated form of β-catenin in subsets of chondrocytes using a mouse line with the exon 3 of *β-catenin* floxed (*Catnb^Ex3^*) [24]. This mouse line was crossed with a tamoxifen (TM) inducible *Cre* line, *Col2a1-CreER* [25]. As the tamoxifen induced Cre activity is not robust in all cells, the activated form of β-catenin is only expressed in a subset of chondrocytes in the *Catnb^Ex3/+^; Col2a1-CreER* embryos following TM injection. Since *Lef1* is a transcription target of Wnt/β-catenin signaling [16], in these subset of chondrocytes, *Lef1* expression was upregulated (Fig. 4A). In addition, bone formation was increased in the TM injected *Catnb^Ex3/+^; Col2a1-CreER* embryos indicated by von Kossa staining (Fig. 4A), suggesting that Wnt/ β-catenin signaling in chondrocytes may also promote bone formation in the perichondrium non-cell autonomously. Interestingly, Safranin O staining and *Col2a1* expression was significantly downregulated in the chondrocytes that expressed the activated form of β-catenin (Fig. 4A). However, these cells did not ectopically express *Col10a1*, a marker for hypertrophic chondrocytes (Fig. 4A). This is in sharp contrast to the normal process of chondrocyte hypertrophy in which *Col2a1* expression is turned off, while *Col10a1* expression is switched on. This is also drastically different from the *Pthr1^−^* ^/*−*^ clones in cartilage [23], in which loss of PTHrP signaling led to cell autonomous expression of *Col10a1*. In addition, while PTHrP signaling does not affect the determination of chondrocyte lineage [26], [27], activated Wnt signaling in the developing cartilage can change chondrocyte identity to the ones that resemble cells found in the joint interzone or joint [28]. These data suggest that activated β-catenin signaling acts cell autonomously to control chondrocyte identity, but non-cell autonomously in regulating chondrocyte hypertrophy. Among the secreted signaling molecules that can regulate chondrocyte hypertrophy, Growth differentiation factor 5 (*Gdf5*) and *Bmp2* expression are upregulated by Wnt/β-catenin signaling [28]–[30]. To address the potential role of these genes, we examined Bmp signaling activities in our mutant mice (Fig. 4B). In and around chondrocytes that express activated β-catenin, we observed stronger phosphorylation of Smad1, 5 and 8, indicating increased Bmp signaling [31]. As Bmp/Gdf5 signaling has been shown to promote chondrocyte hypertrophy [32]–[34], Gdf5 and Bmp2 are likely to participate in mediating the non-cell autonomous function of Wnt/β-catenin signaling in chondrocyte hypertrophy.

## Discussion

We report here that during mouse embryonic cartilage development, Wnt/β-catenin signaling controls chondrocyte hypertrophy and final maturation by two distinct mechanisms. Wnt/β-catenin signaling regulates initiation of chondrocyte hypertrophy by antagonizing PTHrP signaling, whereas it acts independently of PTHrP signaling in controlling the final maturation of hypertrophic chondrocytes (Fig. 4C). Our results indicate that chondrocyte hypertrophy and final maturation are two separate developmental events that are regulated by distinct signaling interactions.

Our data suggests that delayed chondrocyte hypertrophy in the *Catnb^c/c^; Col2a1-Cre* mutant embryo is due to upregulated PTHrP signaling, not enhanced *PTHrP* expression. As β-catenin is required for activating the expression of downstream targets of the canonical Wnt signaling pathway, a possible scenario is that one of the transcription targets of the canonical Wnt signaling pathway in the chondrocytes suppresses the PTHrP signaling activity. Alternatively, the β-catenin protein may directly suppress PTHrP signaling activity by interacting with PTHrP signaling components. Interaction of β-catenin with components of other signaling pathways may be an important mechanism underlying Wnt signaling cross regulation with other pathways. For instance, a LEF/TCF independent regulatory role of β-catenin in cell lineage determination has been demonstrated during pituitary gland development, in which β-catenin binds a specific homeodomain factor, Prop1, to activate expression of the critical lineage-determining transcription factor, Pit1 [35]. However, if β-catenin inhibits PTHrP signaling by directly binding PTHrP signaling component(s), one may predict that PTHrP receptor and activated β-catenin both play a cell-autonomous role in chondrocyte hypertrophy. Although the PTHrP receptor acts cell autonomously in regulating chondrocyte hypertrophy [23], ectopic chondrocyte hypertrophy is not detected in chondrocytes expressing activated β-catenin as these cells have lost their chondrocyte identity because they express neither *Col2a1* nor *Col10a1*. Interestingly, PTH activation of Pthr1 has been shown to activate the Wnt/β-catenin signaling directly by stabilizing β-catenin protein in osteoblasts [36]. However, in chondrocytes, it is unlikely that PTH/PTHrP signaling regulates chondrocyte hypertrophy through β-catenin as loss of PTH/PTHrP and Wnt/β-catenin signaling leads to opposite phenotypes.

Wnt/β-catenin signaling has two distinct roles in cartilage development. First, it inhibits chondrocyte cell lineage determination and maintenance [28]. Second, it promotes chondrocyte hypertrophy by inhibiting PTHrP signaling. However, such inhibition is likely to be incomplete. In addition, Wnt/β-catenin signaling may exert distinct functions in a dose dependent manner. It is conceivable that the two functions of Wnt/β-catenin signaling are also dose-dependent: Strong Wnt/β-catenin signaling (i.e., expression of activated β-catenin) inhibits chondrocyte cell fate determination and maintenance whereas weaker Wnt/β-catenin signaling promotes chondrocyte hypertrophy by reducing PTHrP signaling activities. Our results suggest that Wnt/β-catenin signaling may also interact with PTHrP signaling indirectly through a secondary signaling pathway. To this end, it will be interesting to further investigate genetically whether Gdf5/Bmp signaling control initiation chondrocyte hypertrophy by antagonizing PTHrP signaling and whether Gdf5/Bmp signaling also mediates the role of Wnt/β-catenin signaling in hypertrophic chondrocyte maturation.

## Materials and Methods

### Mice generation and Genotyping

Embryos were generated by crosses between mice carrying heterozygous *PTHrP* null allele *PTHrP^+/−^* [26], *β-catenin* conditional allele *Catnb^c/c^* or gain-of-function allele *Catnb^ex3^*, and a transgene *Cre* which is under the regulation of *Col2a1* promoter and enhancer. Embryos were collected at 14.5, 15.5 and 16.5 dpc and embryos were analyzed as described. Genotyping for the *Col2a1-Cre* transgene and *β-catenin* alleles were performed as previously described [9].

### Skeletal Preparation

Alizarin red and Alcian blue staining of skeletons was conducted as previously described [12]. Embryos were eviscated, fixed for 48 hours in ethanol and then transferred to acetone for 24 hours. Embryos were stained overnight at 37°C followed by 2 additional days at room temperature. Then embryos were cleared in 1% KOH and stored in 80% glycerol.

### Histology and in situ Hybridization

Limbs were fixed by 4% paraformaldehyde at 4°C overnight, embedded in paraffin and sectioned at 6 µm. Sections were stained by Safranin O and Von Kossa method. In situ hybridization was performed as previously described [8]. ^35^S-lablled riboprobes of *Col 2a1, Col 10a1, Ihh, Pthr1, PTHrP, Opn, Mmp13, Mmp9* and *Vegf* were synthesized as previously [9], [12] and applied to limb sections. For immunnohistochemistry, primary antibodies used include anti β-catenin mouse monoclonal IgG (Transduction Lab) at 1∶50, anti-phospho-Smad1/5/8 rabbit polyclonal IgG (Cell signaling) at 1∶100 and anti-RANKL mouse monoclonal IgG (IMGENEX) at 1∶50. The signal was detected using FITC-conjugated secondary antibodies (Molecular Probes) or ABC kits (Vector Laboratories).

### Tamoxifen preparation and injection

60 mg/ml Tamoxifen (Sigma) was dissolved in corn oil (Sigma) and sonicated until the solution became clear. The solution was filtered and 0.05 ml was injected intraperitoneally into the pregnant female mice at 13.5 dpc and the embryos were harvested at 17.5 dpc.

## Supporting Information

Figure S1 Analysis of chondrocyte hypertrophy in vertebral skeletons. Vertebral skeletons were visualized by Alcian Blue / Alizarin Red staining at E14.5. Mineralization was greatly reduced in the ribs and vertebral bodies in both the Catnbc/c; Col2a1-Cre single mutant and the PTHrP−/−; Catnbc/c; Col2a1-Cre double mutant. Chondrocyte hypertrophy was revealed by Safranin O staining and the expression of Col2a1 and Col10a1 in sections of vertebral bodies at E14.5. Compared to the Catnbc/c; Col2a1-Cre single mutant, chondrocyte hypertrophy in the PTHrP−/−; Catnbc/c; Col2a1-Cre double mutant was much accelerated and similar to that in PTHrP−/− single mutant embryos indicated by the expression of Col10a1.
(3.47 MB TIF)

Figure S2 Analysis of chondrocyte proliferation in PTHrP and β-catenin mutant embryos. (A) BrdU-labeled chondrocytes were detected by immunohistochemistry on sections of distal tibia at 16.5dpc. Zone I cells are resting chondrocytes and Zone II are columnary proliferating chondrocytes. Zone I and II were greatly reduced and the difference between them was not clear in the PTHrP−/− single mutant and PTHrP−/−; Catnbc/c; Col2a1-Cre double mutant embryos. The entire proliferating region was marked as Zone I and II. (B) The percentage of BrdU labeled chondrocytes was counted from four different samples of each genotype and the average with standard deviations are shown. Significant difference with p<0.05 is shown.
(2.47 MB TIF)

Figure S3 Analysis of osteoblast differentiation in PTHrP and β-catenin mutant embryos. Consecutive sections of developing humerus at E14.5 and E16.5 were examined by in situ hybridization with indicated probes. (A) At E14.5, expression of early osteoblast marker Runx2 and Osx in PTHrP−/−; Catnbc/c; Col2a1-Cre double mutant embryos was accelerated to the same level as that in PTHrP−/− mutant embryos. (B) At E16.5, expression of Runx2 and Osx in double mutant embryos was accelerated whereas Osc expression was still delayed compared to that in PTHrP−/− single mutant embryos.
(4.01 MB TIF)

Figure S4 Analysis of RANKL expression in PTHrP and β-catenin mutant embryos. Proximal tibia sections at E16.5 were examined by immunohistochemistry with a RANKL monoclonal antibody. The expression of RANKL by osteoblasts is indicated by an arrow. RANKL expression in mature hypertrophic chondrocytes was very low and slightly increased in the Catnbc/c; Col2a1-Cre mutant but not in the PTHrP−/− and PTHrP−/−;Catnbc/c;Col2a1-Cre mutant embryos.
(3.75 MB TIF)
