# Supplementary figures and images for: The Wnt/β-Catenin Pathway Interacts Differentially with PTHrP Signaling to Control Chondrocyte Hypertrophy and Final Maturation
Source: PLoS One. 2009 Jun 26;4(6):e6067. doi: 10.1371/journal.pone.0006067 (PMC2698152; doi:10.1371/journal.pone.0006067)

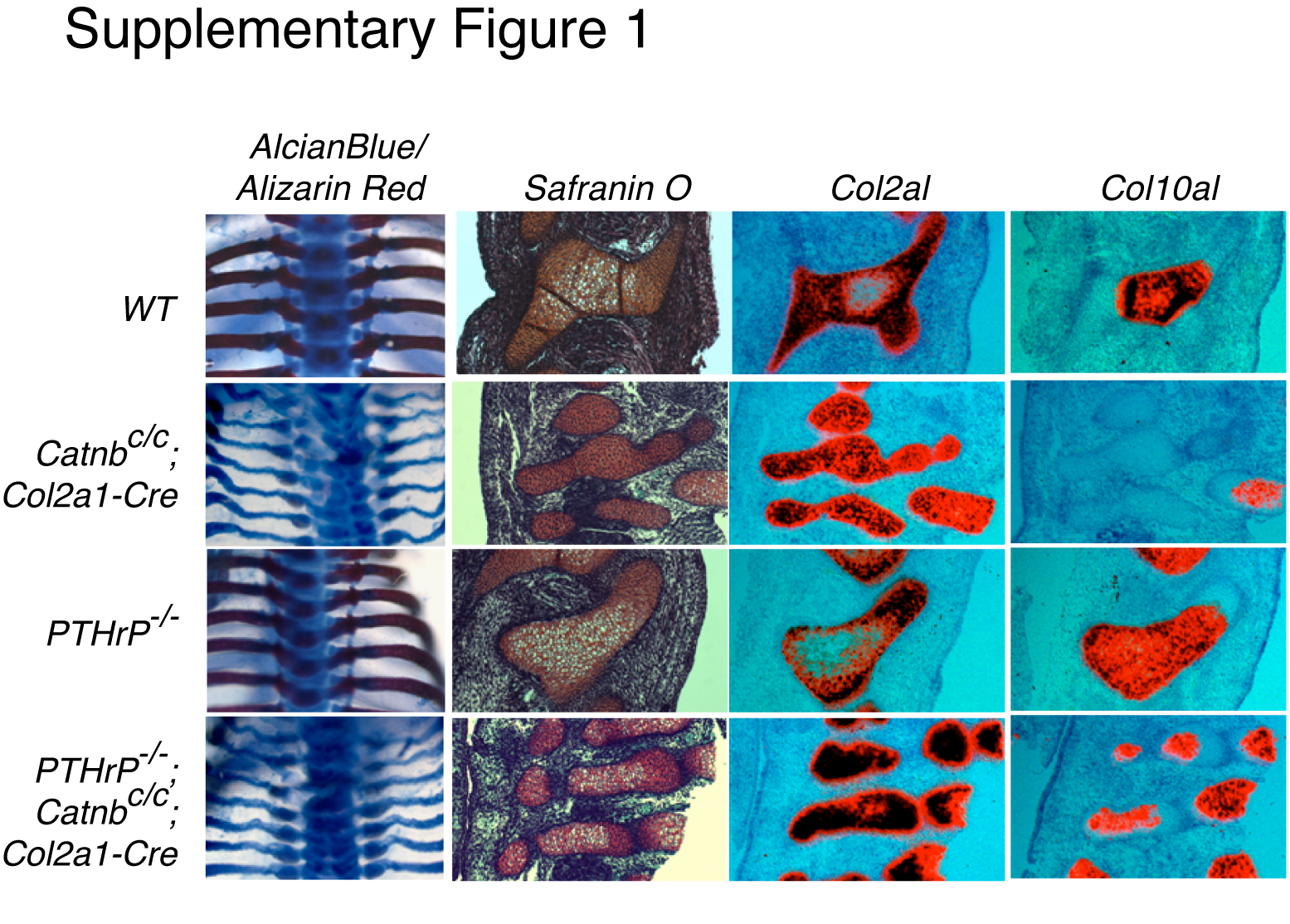

Supplement: Figure S1 — Analysis of chondrocyte hypertrophy in vertebral skeletons. Vertebral skeletons were visualized by Alcian Blue / Alizarin Red staining at E14.5. Mineralization was greatly reduced in the ribs and vertebral bodies in both the Catnbc/c; Col2a1-Cre single mutant and the PTHrP−/−; Catnbc/c; Col2a1-Cre double mutant. Chondrocyte hypertrophy was revealed by Safranin O staining and the expression of Col2a1 and Col10a1 in sections of vertebral bodies at E14.5. Compared to the Catnbc/c; Col2a1-Cre single mutant, chondrocyte hypertrophy in the PTHrP−/−; Catnbc/c; Col2a1-Cre double mutant was much accelerated and similar to that in PTHrP−/− single mutant embryos indicated by the expression of Col10a1. (3.47 MB TIF) [file pone.0006067.s001.tif]

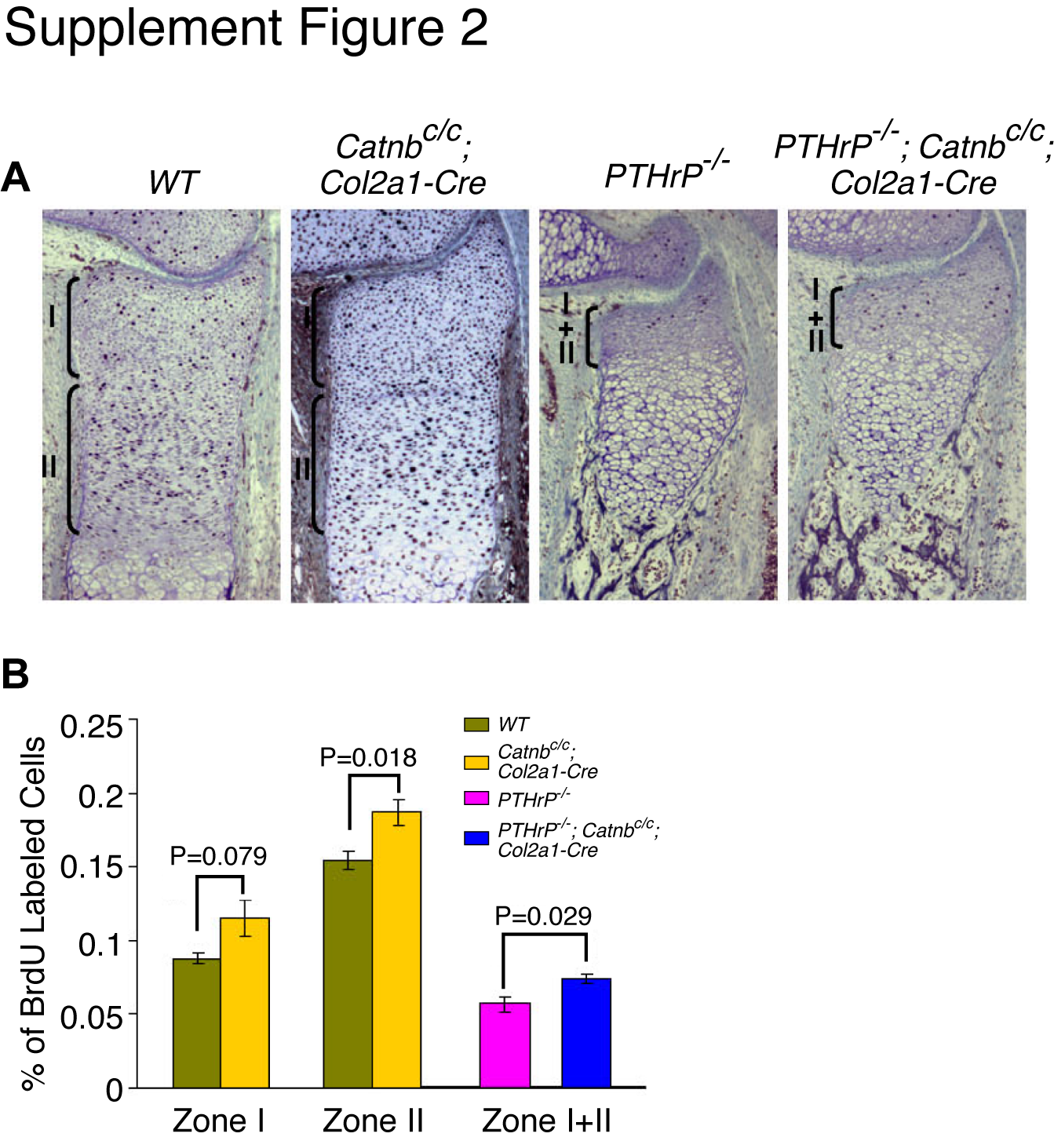

Supplement: Figure S2 — Analysis of chondrocyte proliferation in PTHrP and β-catenin mutant embryos. (A) BrdU-labeled chondrocytes were detected by immunohistochemistry on sections of distal tibia at 16.5dpc. Zone I cells are resting chondrocytes and Zone II are columnary proliferating chondrocytes. Zone I and II were greatly reduced and the difference between them was not clear in the PTHrP−/− single mutant and PTHrP−/−; Catnbc/c; Col2a1-Cre double mutant embryos. The entire proliferating region was marked as Zone I and II. (B) The percentage of BrdU labeled chondrocytes was counted from four different samples of each genotype and the average with standard deviations are shown. Significant difference with p<0.05 is shown. (2.47 MB TIF) [file pone.0006067.s002.tif]

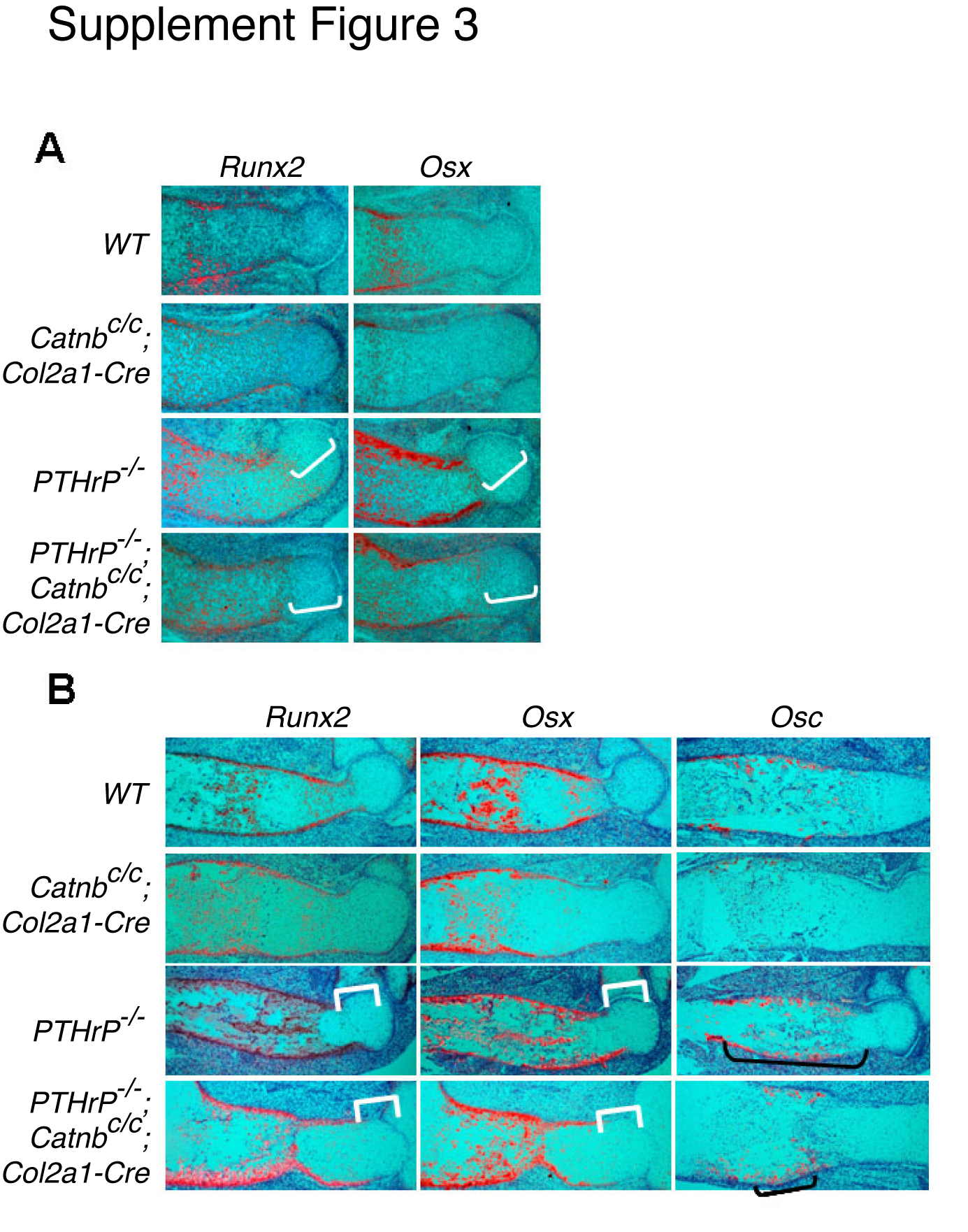

Supplement: Figure S3 — Analysis of osteoblast differentiation in PTHrP and β-catenin mutant embryos. Consecutive sections of developing humerus at E14.5 and E16.5 were examined by in situ hybridization with indicated probes. (A) At E14.5, expression of early osteoblast marker Runx2 and Osx in PTHrP−/−; Catnbc/c; Col2a1-Cre double mutant embryos was accelerated to the same level as that in PTHrP−/− mutant embryos. (B) At E16.5, expression of Runx2 and Osx in double mutant embryos was accelerated whereas Osc expression was still delayed compared to that in PTHrP−/− single mutant embryos. (4.01 MB TIF) [file pone.0006067.s003.tif]

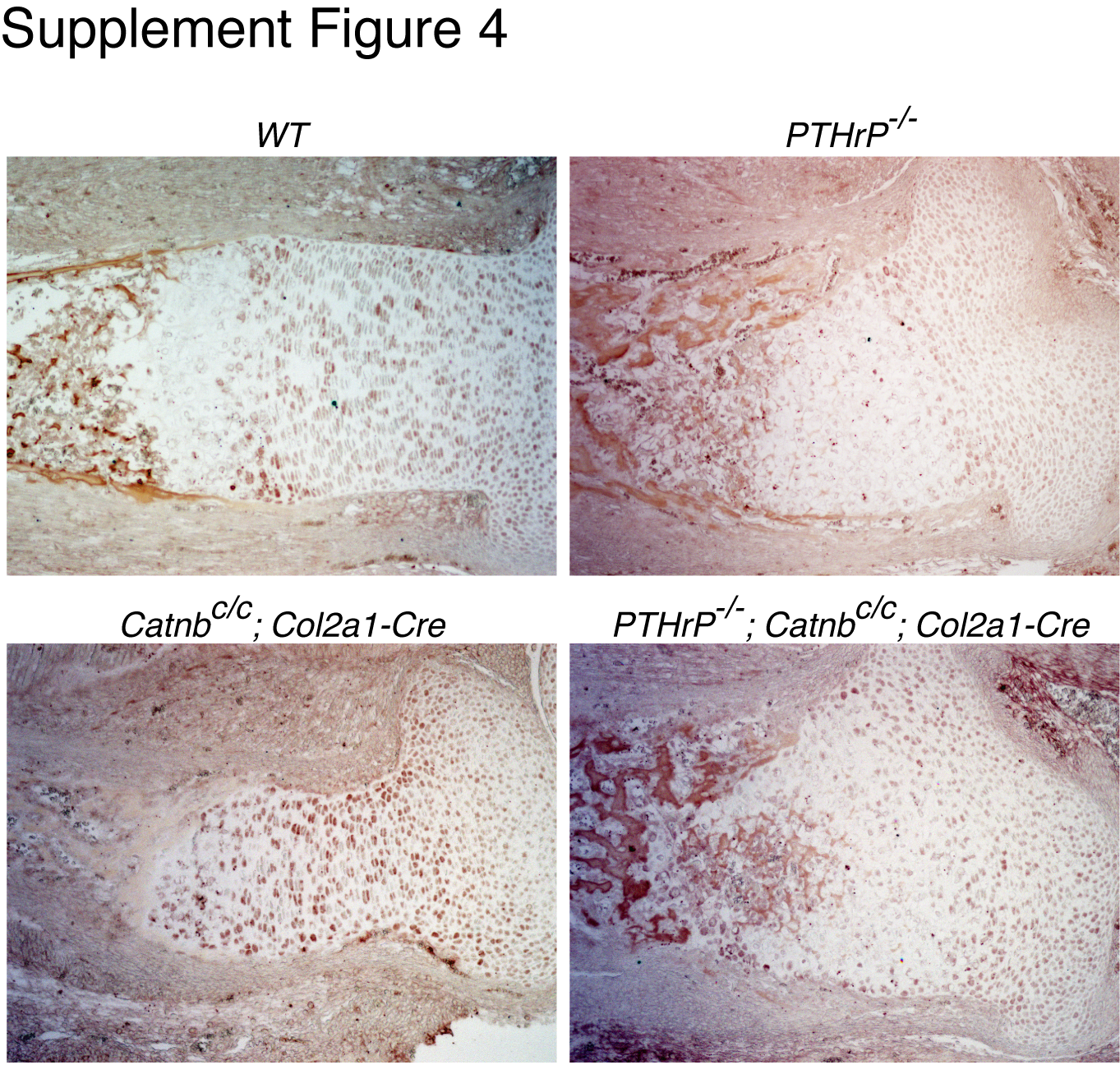

Supplement: Figure S4 — Analysis of RANKL expression in PTHrP and β-catenin mutant embryos. Proximal tibia sections at E16.5 were examined by immunohistochemistry with a RANKL monoclonal antibody. The expression of RANKL by osteoblasts is indicated by an arrow. RANKL expression in mature hypertrophic chondrocytes was very low and slightly increased in the Catnbc/c; Col2a1-Cre mutant but not in the PTHrP−/− and PTHrP−/−;Catnbc/c;Col2a1-Cre mutant embryos. (3.75 MB TIF) [file pone.0006067.s004.tif]
